# Immune checkpoint inhibitors as a real hope in advanced urothelial carcinoma

**DOI:** 10.4155/fsoa-2018-0033

**Published:** 2018-10-04

**Authors:** Emmanuel Seront, Gaëtan Catala, Alexandre Dermine, Sarah Lejeune, Stephane Rysselinck

**Affiliations:** 1 Department of Medical Oncology, Hopital de Jolimont, 7100 Haine Saint Paul, Belgium; 2 Department of Urology, Hopital de Jolimont, 7100 Haine Saint Paul, Belgium

**Keywords:** atezolizumab, avelumab, durvalumab, gene signature, immune checkpoint inhibitors, immunotherapy, nivolumab, pembrolizumab, programmed death ligand-1 (PD-L1), urothelial carcinoma

## Abstract

Metastatic urothelial cancer is an aggressive disease associated with a poor prognosis. In the first-line setting, platinum-based chemotherapy is the standard of care but resistance rapidly occurs. After failure of platinum-based therapy and in cisplatin-ineligible patients, therapeutic options are limited. Malignant cells evolve mechanisms to evade immune recognition, including the expression of cell-surface molecules, named immune checkpoints, on tumor and tumor-specific lymphocytes. Immunotherapy, by targeting these checkpoints, represents a new tool to improve the patient outcome in advanced urothelial carcinoma (UC). Recently, the US FDA approved, in a short time, several immune checkpoint inhibitors in metastatic UC, both after failure of platinum-based therapy and in first-line setting in cisplatin-ineligible patients. This article aims to review the place of immunotherapy in advanced UC.

## Lay abstract

Urothelial carcinoma is an aggressive disease and therapeutic options are limited in patients with advanced stage who are refractory to chemotherapy. Immunotherapy represents a milestone for these patients; different immune checkpoint inhibitors have shown significant activity in advanced urothelial carcinoma and are currently available both in the second-line metastatic setting (after failure of platinum-based therapy) and in the first-line setting in cisplatin-ineligible patients. Furthermore, these agents are better tolerated than chemotherapy. PD-L1 expression is not an ideal biomarker and further research is evaluating innovative methods to facilitate selection of patients who are most likely to benefit from these agents.

Bladder cancer (BC) is the seventh most common cancer worldwide with more than 430,000 newly diagnosed cases, and approximately 165,000 deaths, each year [1]. Urothelial carcinoma (UC) accounts for more than 90% of BC. The majority of patients (around 75%) present with localized and nonmuscle-invasive BC are treated with curative intent; treatments include surgical resection, intravesical chemotherapy and/or intravesical injection of BCG. Conversely, muscle-invasive BC requires a multimodal strategy, including cystectomy and chemotherapy [2]. Despite this aggressive management, more than 50% of muscle-invasive BC patients develop metastases with a poor prognosis. Cisplatin-based chemotherapy is the only treatment that significantly improves survival in first-line metastatic UC (mUC). Two regimens, methotrexate/vinblastine/adriamycin/cisplatin and cisplatin/gemcitabine, have shown greater activity over cisplatin alone in the first-line setting with objective response rates (ORR), progression-free survival (PFS) and overall survival (OS) in the range of 40–49%, 7.7–10 months and 12.5–14.8 months, respectively [3]. Strategies, such as adding paclitaxel to cisplatin [4] or increasing the dose intensity of the methotrexate/vinblastine/adriamycin/cisplatin regimen (administration every 14 days with granulocyte colony-stimulating factor support) [5], have also been evaluated and, despite increases in ORR and PFS, no survival advantage was observed. Due to its better tolerability and safety profile, the cisplatin/gemcitabine combination remains the standard of care for patients with mUC in a first-line setting.

A proportion of patients are ineligible for cisplatin due to poor performance status and renal failure, and are treated with other and less efficient regimens such as carboplatin–gemcitabine [6]. With this regimen, ORR does not exceed 36%, PFS 5 months and OS 9 months, respectively. Platinum resistance rapidly occurs and nearly 80% of cases will relapse. Prognosis is extremely poor after failure of platinum-based chemotherapy; cytotoxic drugs, as single agents or in combination, have shown poor activity in the second-line setting with response rates, median PFS and median OS ranging from 10 to 20%, 2–4 and 6–9 months, respectively [7–9]. Advances in the immuno-oncology field have considerably improved outcome for patients with different cancer types, including UC. In a short time, different immune checkpoint inhibitors (ICIs) have been approved in mUC enlarging the choice for therapeutic options in mUC.

## Rational for immunotherapy in BC

UC is a immune-responsive cancer, as intravesical instillations of BCG has shown to prevent recurrences of high-risk NMIUC, by eliciting a cytotoxic immune response [10]. Immune system is able to detect and eliminate cancer cells, as they exhibit differences in antigenicity from healthy cells. Tumor cells release tumor-associated antigens, named neoantigens that are captured by antigen-presenting cells (APC) through the MHC-I. APC migrate to lymphoid organs, where they activate effector T-cells, which in turn infiltrate tumors, and kill cancer cells. However, malignant cells evolved different mechanisms to evade immune recognition; one such strategy involves the expression of cell-surface molecules, named immune checkpoints, on tumor and tumor-specific lymphocytes, that are able to inhibit activated T-cells. The most commonly investigated immune checkpoints are CTLA-4, PD-1 and PD-L1. Activation of T-cells requires interaction between CD28 on T-cell and B7 on APC. CTLA-4 expressed on T-cell exerts its inhibitory effect by competing with CD28 and by binding to B7, resulting in T-cell inactivation in lymphoid tissues. In the same way, PD-1 is an inhibitory receptor expressed on T-cells. When binding to PD-1, PD-L1 expressed on tumor cells transmits an inhibitory signal into T-cells [11–13].

ICIs are monoclonal antibodies that target immune checkpoints, and thereby disrupt the inhibitory signals and reactivate immune system.

**Figure 1.**
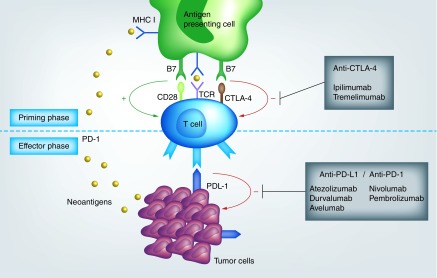
**Immunotherapy agent mechanisms.**

Two monoclonal antibodies targeting CTLA-4 have been developed: ipilimumab and tremelimumab. The most studied PD-L1 inhibitors include atezolizumab, durvalumab and avelumab, are PD-L1 inhibitors; nivolumab and pembrolizumab are PD-1 inhibitors (Figure 1).

UC appears to be as a good candidate for immunotherapy. In a retrospective analysis, patients with increased tumor-infiltrating CD8^+^ lymphocytes (TILs) within advanced UC (pT2, pT3 or pT4) have better disease-free survival and OS than patients with similar-staged UC and fewer intratumoral CD8 TILs, suggesting that intratumoral TIL infiltration is associated with better outcome [14]. Moreover, UC carries the third highest mutation rate of all studied cancers, resulting in production of high amount of neoantigens, which is required for antigenicity and effective immune response [15]. Furthermore, bladder tumors and infiltrating immune cells (ICs) exhibiting increased expression of PD-L1 and PD-1 are associated with poorer outcomes. Levels of PD-L1 expression in NMIUC have been correlated with BC higher-stage, higher frequencies of postoperative recurrence and poorer survival [16].

This review focuses on the different agents that are currently approved in mUC and highlights promising therapeutic strategies in this field.

### Immune checkpoint inhibitors as second-line agent in mUC

In May 2016, the PD-L1 inhibitor atezolizumab was the first US FDA-approved ICI as second-line treatment for inoperable locally advanced and mUC progressing during or after platinum-based chemotherapy. This approval was based on the results of the nonrandomized Phase II IMvigor210 trial Cohort 2. This cohort enrolled 310 patients with inoperable locally advanced or mUC whose disease had progressed following platinum-based chemotherapy. All these patients were stratified according to tumor-infiltrating IC PD-L1 expression in three subgroups: IC0 (<1%), IC1 (≥1% but <5%) and IC2/3 (≥5%). 20% of patients received ≥ 3 previous chemotherapy regimen. Atezolizumab (1200 mg IV q3w) resulted in an ORR (primary end point) of 16%, including 7% of complete response (CR) in all patients; the ORR reached up to 28%, including 15% CR in IC2/3 patients. Up to 10% of responses was also seen in PD-L1-negative patients. Most responses were rapid, with a median time to response of 2.1 months. The responses tended to be durable; the median duration of response (DOR) was not reached after a medium of 17.5 months of follow-up and 84% of response were ongoing. The median OS was 7.9 months for all patients and 11.9 months in IC2/3 patients, and the 1-year OS rate was 37 and 50%, respectively. These responses and survival results were better than those observed with historical second-line agents such as vinflunine, ifosfamide or paclitaxel [7–9]. Atezolizumab, as the majority of ICIs, was well tolerated; the most common treatment-related adverse events (TRAEs) with atezolizumab in the overall population were fatigue (31%) and nausea (14%). About 16% of patients experienced Grade 3/4 TRAEs, including 5% of immune-related AEs, reflecting a better toxicity profile compared with chemotherapy [17]. These promising results led to the randomized Phase III IMvigor211 trial that compared atezolizumab with standard second-line chemotherapy (vinflunine, paclitaxel, docetaxel) in 931 mUC patients after failure of platinum-based chemotherapy. The primary efficacy end point OS was to be tested in a successive fashion in study populations defined by IC PD-L1 expression, starting with high (IC2/3) PD-L1 expression, followed by those with any level of PD-L1 expression (IC1/2/3), and followed by the overall study population (intention-to-treat [ITT]). Statistical significance needed to be achieved in the IC2/3 population in order to evaluate the ITT population for statistical significance, and similarly achieved in the IC1/2/3 population in order to evaluate the overall study population for statistical significance. Surprisingly, atezolizumab failed to demonstrated improved OS compared with chemotherapy, with, in high PD-L1 patients, a median OS (mOS) of 11.1 months compared with 10.6 months (hazard ratio (HR): 0.87; p = 0.41) and a 1-year OS rate of 46% compared with 41%, respectively. A moderate but significant difference in OS was observed in the ITT analysis for all patients treated with atezolizumab (8.6 months) compared with chemotherapy (8 months; HR: 0.85; p = 0.038). These perplexing results could be explained by the fact that the OS in the chemotherapy arm, and particularly in the vinflunine arm, appeared better than study design assumptions. One hypothesis is that the PD-L1-positive cohort was a smaller sample size and insufficiently powered to address the benefit in median OS (mOS) for this cohort. The use of archival specimens may have confounded the true assessment of PD-L1-expression at the time of study entry. PD-L1 expression could also appear as a prognostic factor and selection of PD-L1-positive patients could have potentially selected patients with better prognosis, explaining a such impressive survival both in atezolizumab and in chemotherapy arm. However, and consistently with Imvigor210 results, the median DOR with atezolizumab was 21.7 months in the overall study population, compared with 7.4 months with chemotherapy, confirming robust antitumoral efficacy of atezolizumab [18]. In light of these results and the better safety profile compared with chemotherapy, atezolizumab appears to be an alternative to chemotherapy in second-line metastatic setting in mUC, although the absence of level 1.

A study of atezolizumab monotherapy or in combination with chemotherapy (atezolizumab ± gemcitabine/carboplatin or cisplatin vs chemotherapy alone) is ongoing investigating among patients with treatment-naive locally advanced or mUC (NCT02807636).

In February 2017, the FDA approved nivolumab, a monoclonal anti-PD1, as second therapy for patients with mUC. Approval was based on the Phase II single-arm CheckMate 275 study that enrolled 265 previously treated mUC patients. The single-arm, open-label, Phase II CheckMate 275 study evaluated the activity and efficacy of nivolumab (3 mg/kg IV q2w) in 265 previously treated patients with mUC. Nivolumab resulted in an ORR of 19.6% for the total population. The response rate was related to tumor PD-L1 expression; 28.4% for patients with tumor PD-L1 expression ≥5%, 23.8% for patients with tumor PD-L1 expression ≥1% and 16.1% for patients with low PD-L1 expression (<1%). Median DOR was not reached and 77% of responses were ongoing at the time of analysis. The median OS was 8.74 months in all patients and increased to 11.3 months in PD-L1 ≥1% patients compared with 5.95 months in PD-L1 less than 1% patients. Nivolumab was well tolerated, with 18% of Grade 3–4 TRAEs (mostly fatigue and diarrhea). The most common immune-related AEs (any grade) were skin (17%) and endocrine (14%). Three deaths were attributed to treatment (pneumonitis, acute respiratory failure and cardiovascular failure) [19]. At the American Society of Clinical Oncology Genito-Urinary Symposium 2018, the long-term data on the nivolumab arm of the initial Phase I/II CheckMate 032 that was first published in 2016 was presented [20]. At 2 years of follow-up, the ORR is 25.6%, regardless of PD-L1 expression, and 65% of patients who initially responded are still enjoying a durable response at 2 years. The 12-month OS reaches 46% and 2-year OS 37%, with a median OS of 10 months, confirming the durable response pattern observed with ICIs. No new toxicity signal was reported [21].

In May 2017, the FDA approved the anti-PD1 antibody pembrolizumab after failure of platinum-based therapy in UC; pembrolizumab is the first agent to demonstrate a survival benefit in mUC in the KEYNOTE-045 trial, a randomized Phase III trial that compared efficacy of pembrolizumab (200 mg q3w) with chemotherapy (docetaxel, paclitaxel or vinflunine) in 524 patients with mUC who progressed during or after a platinum-based chemotherapy. After median follow-up of 14.1 months, OS in all patients was significantly improved with pembrolizumab compared with chemotherapy (10.3 vs 7.4 months, respectively; p = 0.002;). PFS was not significantly different between pembrolizumab and chemotherapy in all patients. The ORR was significantly better with pembrolizumab than with chemotherapy (21.1 vs 11.4%). Responses were durable with, at the time of data analysis, 18.4% of patients still receiving pembrolizumab compared with only 1.2% receiving chemotherapy. Interestingly, PD-L1 status, based on a ‘combined positive score’, which was measured as the percentage of PD-L1-positive immune and tumor cells compared with the number of tumor cells, was not associated with a better OS, PFS or ORR in pembrolizumab arm. In the pembrolizumab arm, high PD-L1 expression was even associated with a lower OS (8 months) compared with that of all pembrolizumab-treated patients, suggesting that this biomarker could highlight a poor-prognosis group. Therefore, PD-L1 status using the 10% combined positive score threshold should not be used to select which patients with previously treated mUC should receive pembrolizumab. Pembrolizumab was better tolerated than chemotherapy, with Grade 3–4 toxicities not exceeding 15%. The most common immune-mediated AEs were pruritus (20%), thyroid abnormalities (9%), pneumonitis (4%) and colitis (2%) [19]. The 2-year follow-up confirmed the safety and the superiority of pembrolizumab compared with chemotherapy with a longer median DOR (not reached [1.6–30.3 months] compared with 4.4 months [1.4–29.9]), and a greater proportion of responses lasting ≥12 months (68 vs 35%, respectively) [23]. Pembrolizumab represents thus a level 1 evidence in second-line setting, after failure of platinum-based therapy in mUC.

Other trials are currently ongoing; pembrolizumab is being investigated in combination with docetaxel or gemcitabine (NCT02437370).

In May 2017, two new, selective, high-affinity monoclonal antibodies against PD-L1, durvalumab and avelumab, received FDA-approval as a treatment for inoperable or metastatic urothelial BC patients progressing on platinum-based treatment. The decision for durvalumab approval was based on the Phase I/II study (NCT01693562), which evaluated its safety, tolerability and antitumor activity as monotherapy (10 mg/kg IV q2w) in 191 patients with advanced UC; 95% had progressed while receiving or after receiving a platinum-based therapy or within 12 months of receiving therapy in a neoadjuvant or adjuvant setting. Patients were stratified based on PD-L1 expression on tumor or ICs, high (≥25%) versus low (<25%); this cutoff was defined because it seemed to enrich for response, based on review of PD-L1 expression in the first 20 enrolled patients who were followed for a minimum of 12 weeks [24]. The ORR was 17.8% including 3% CR in the entire population; ORRs were 27.6% in PD-L1 high patients and 5.1% in PD-L1 low or negative patients. Responses occurred early with a median time to response of 1.41 months and were durable with a median DOR that was not reached at data cutoff (range: ≥0.9 to ≥19.9 months). The disease control rates were numerically greater in PD-L1 high versus low or negative subgroups (44.9 vs 21.5%) in the entire population. Median PFS and OS were 1.5 and 18.2 months, respectively; the 1-year OS rate was 55%. Grade 3/4 TRAEs occurred in 6.8%, including 2% Grade 3/4 immune-mediated AEs. The median PFS was 1.5 months in the entire population and was higher in PD-L1 high patients (2.1 months) compared with low or negative patients (1.4 months). The median OS was 18.2 months in the entire population and was also higher in PD-L1 high (20.0 months) compared with low or negative patients (8.1 months). The OS rates at 6, 9 and 12 months were 64, 57 and 55%, respectively, in the entire population, which is superior to the second-line cytotoxic regimen that are currently used in clinical practice [25]. The Phase III DANUBE trial that enrolled 1004 mUC patients is comparing standard chemotherapy with durvalumab alone and with the combination of durvalumab and the CTLA-4 inhibitor tremilimumab; OS is the primary end point (NCT02516241).

Compared with durvalumab and atezolizumab, avelumab can, in addition to PD-L1 inhibition, induce antibody-dependent cell-mediated cytotoxicity, which results in a direct lysis of tumor cells. The JAVELIN solid tumor trial (NCT01772004) is investigating safety, tolerability and clinical activity of avelumab (10 mg/kg IV q2w) in patients with locally advanced or metastatic solid tumors. In this Phase Ib dose-expansion cohort, 44 patients with mUC received avelumab after failure of platinum-based therapy. Patients were categorized based on tumor cell PD-L1 expression with a cutoff of ≥5%. Avelumab resulted in a confirmed ORR of 18.2%, including 11.4% CR, and a disease control rate of 52.3%. The median DOR was not reached at data analysis but ongoing responses for ≥48 weeks were observed in 70% of responders. The confirmed ORR increased to 53.8% in PD-L1-positive tumors and was 4.2% in PD-L1-negative tumors. The median PFS was 11.6 weeks and the median OS was 13.7 months with a 1-year OS rate of 54.3% in the entire population. Grade 3–4 TRAEs occurred in 6.8% patients [26].

A second cohort enrolled additional patients with mUC and a pooled analysis from these two cohorts included 249 avelumab-treated patients. An update analysis showed that in 161 postplatinum patients with ≥6 months of follow-up, ORR was 17.4%, including 6.2% CR with a disease control rate of 39.8%. Response was ongoing in 82.1% of responders at data cut with a median DOR that was not reached. Regarding the PD-L1 expression, the ORR was higher in PD-L1 positive (25%) compared with PD-L1-negative subgroup (14.7%). The median PFS was 6.6 weeks and the median OS was 7.4 months with a 6-month OS rate that reached 54.9%. Treatment was well tolerated, with only 8.4% Grade-4 TRAEs and one treatment-related death (pneumonitis) [27].

The Phase III JAVELIN Bladder 100 study (NCT02603432) is ongoing to evaluate avelumab plus best supportive care versus best supportive care alone as first-line maintenance in patients with locally advanced or mUC whose disease did not progress after platinum-containing chemotherapy.

## ICIs as first-line treatment in cisplatin-ineligible patients

Two agents are currently approved in first-line metastatic setting in cisplatin-ineligible patients, as they appear superior to historical carboplatin–gemcitabin regimen, despite the absence of randomized study.

Atezolizumab was approved in April 2017 as first-line agent in cisplatin-ineligible patients. Cohort 1 of the IMvigor210 study enrolled 119 patients who were ineligible for cisplatin-based chemotherapy and who had received no prior chemotherapy in metastatic setting. Atezolizumab resulted in a 24% ORR in all patients, including 7% CR; 75% of response was durable at 14.4 month of follow-up. Median OS reached 15.9 months and the 1-year OS was 57%, which is higher than the 9.3 months OS and 37% 1-year OS observed with historical carboplatin-gemcitabine regimen [6]. Conversely to cohort 1, high PD-L1 expression was not associated with high ORR or OS [28]. The Phase III IMvigor130 (NCT02807636) study is ongoing to confirm the benefit of atezolizumab in this population.

In June 2017, pembrolizumab received accelerated approval for the treatment of mUC cisplatin-ineligible patients in a first-line setting. This was based on the Phase II trial KEYNOYTE-052 that evaluated pembrolizumab as first-line agent in 370 ciplatin-ineligible patients (42% of patients with ECOG 2, 49% with renal dysfunction and 10% with both). The primary end point ORR reached 24%, including 5% CR and stable disease was achieved in 23%, resulting in clinical benefit of 47%. As of data cutoff, median DOR was not yet reached (95% CI: 9 months – not reached). 83% of responses were ongoing as of data cutoff, with 78% (95% CI: 63–87) of responses lasting at least 6 months. The median PFS was 2 months (95% CI: 2–3), with a 6-month PFS of 30%. The 6-month OS was 67% (95% CI: 62–73). Pembrolizumab was well tolerated; 62% of patients had a TRAE, including 16% with TRAEs of Grade 3 or worse. The most common grade ≥3 TRAEs were fatigue (2%), alkaline phosphatase increase (1%), colitis (1%) and muscle weakness (1%) [29]. Selection of patients appears very important when initiating ICIs. In the KEYNOTE-052 trial, ORR reached 47% in patients with lymph nodes only, while it fell to 23% in visceral disease, suggesting that chemotherapy could also be an attractive way in some cases, such as bulky and rapidly progressive diseases. In first-line setting, in cisplatin-ineligible patients, pembrolizumab and atezolizumab represent thus an alternative to the carboplatin-based regimen. These trials are summarized in Table 1.

**Table 1.** **Key trials in urothelial carcinoma.**

**Study**	**Arm**	**n**	**ORR**	**OS (months)**	**Treatment-related adverse events (%)**
Second-line setting, after failure of platinum-based therapy					

Phase II single-agent IMvigor210 [17]	Atezolizumab	310	All pts = 16%, CR: 7% High PD-L1 = 28%, CR: 15%	All pts = 7.9 1-year OS = 37% High PD-L1 = 11.9 1-year OS = 50%	Any grade = 66% Grade 3–4 = 16%

Phase III randomized IMvigor 211 [18]	Atezolizumab	467	All pts = 14%, CR: 4% High PD-L1 = 23%	All pts = 8.9 1-year OS = 40% High PD-L1 = 11.1	Any grade = 70% Grade 3–4 = 20%

Vinflunine or paclitaxel or docetaxel	464	All pts = 15%, CR: 4% High PD-L1 = 22%	All pts = 8.2 1-year OS = 33% High PD-L1 = 10.6	Any grade = 89% Grade 3–4 = 43%	

Phase II single-agent Checkmate 275 [19,21]	Nivolumab	265	All pts = 19.6% High PD-L1 = 28.4 Low PD-L1 = 16.1%	All pts = 8.7 1-year OS = 41% High PD-L1 = 11.3 Low PD-L1 = 5.95	Any grade = 64% Grade 3–4 = 18%

Phase III randomized Keynote-045 [23]	Pembrolizumab	270	All pts = 21.1%, CR: 7%	All pts = 10.3 High PD-L1 = 8	Any grade = 61.3% Grade 3–4 = 16.5%

Vinflunine or paclitaxel or docetaxel	272	All pts = 11.4%, CR: 3.3%	All pts = 7.4 High PD-L1 = 5.2	Any grade = 90.2% Grade 3–4 = 49.8%	

Phase I/II single-agent [24,25]	Durvalumab	182	All pts = 17% High PD-L1 = 26.3% Low PD-L1 = 4.1%	All pts = 14.1 1-year OS = 50%	Any grade 60.7% Grade 3–4 = 6.8%

Phase Ib single-agent (Javelin) [26,27]	Avelumab	242	All pts = 16.1% High PD-L1 = 25% Low PD-L1 = 14.7%	All pts = 7.4 1-year OS = 54.9%	Any grade = 66.7% Grade 3–4 = 8.4%

First-line setting, in cisplatin-ineligible patients					

Phase II single-agent IMvigor210 cohort 1 [28]	Atezolizumab	119	All pts = 24%, CR: 7% High PD-L1 = 24%	All pts = 15.9 High PD-L1 = 12.3 Low PD-L1 = 19.1	Any grade = 66% Grade 3–4 = 18%

Phase II single-agent Keynote-052 [29]	Pembrolizumab	370	All pts = 29%, CR: 7%	6-month OS = 67%	Any grade = 62% Grade 3–4 = 16%

CR: Complete response; ORR: Objective response rate; OS: Overall survival; PD-L1: Programmed death ligand-1; Pts: Patients.

## The biomarker issue

Data concerning the correlation between ICIs efficacy and PD-L1 expression are heterogenous. Response rates and survival increase with PD-L1 expression in some trials [17–21], but not in other trials [22,23,28]. For example, in the IMvigor210, atezolizumab efficacy increased with PD-L1 expression in cohort 2 (patients receiving atezolizumab after failure of platinum-based therapy), whereas there was no association in cohort 1 (patients ineligible for cisplatin and previously untreated), despite a similar method of PD-L1 detection [22,28]. However, a proportion of PD-L1-negative patients in these trials also benefit from ICIs and other trials showed absence of correlation between response and PD-L1 expression, highlighting limitation of PD-L1 as biomarker. This could be explained by the fact that PD-L1 expression could be dynamic and heterogeneous between primary tumor and metastases. PD-L1 dynamic change was recently evaluated in 27 paired samples of primary and metastasis of UC. The PD-L1 score on tumor cells (TCs) of the primary tumor correlated significantly with the PD-L1 score of the metastasis with statistical homogeneity and equal distribution with a PD-L1 positivity (IC1/2/3) on TCs of 59.3% in both primary tumors and metastases. Conversely, regarding PD-L1 expression on ICs, no significant correlation between primary tumors and metastases was shown with a high discordance rate of 44.4% (PD-L1 positivity tumor vs metastasis: 77.7 vs 44.4%), resulting in significant dynamic changes between primary tumor and metastasis [30]. Whether this dynamic change is related to natural history or to treatments patients received is unknown. Furthermore, detection and interpretation assays of PD-L1 staining are not standardized, particularly regarding the cutoffs and the kind of cells expressing PD-L1. Some studies measured PD-L1 in the tumor, some measure PD-L1 in immune-infiltrating cells and some measure both, all with different PD-L1 antibodies (SP142, 22C3, 28-8 and 5H1). The assays use different cutoffs for positivity, including 1, 5%, and an IHC score based on a sliding range. This lack of standardized PD-L1 testing is an important limitation in the validation of PD-L1 as a predictive biomarker across trials. However, PD-L1 alone could not be sufficient as a valuable predictive tool. The Tumor Cancer Genome Atlas (TCGA) recently identified four molecular UC cluster subsets, according to different genetic signature and outcome (luminal clusters I and II, basal clusters III and IV) [31]. Some clusters have been associated with sensitivity to ICIs and could appear as a complementary tool for ICIs response prediction. In IMvigor210 trial, responses were observed across all TCGA subtypes but the ORR was significantly higher in the luminal cluster II subtype, which was characterized by transcriptional signatures associated with the presence of activated T-effector cells and high IC PD-L1 expression. In contrast, luminal cluster I was associated with low expression of CD8^+^ effector genes, lower PD-L1 IC/TC expression and lower responses to atezolizumab. Basal clusters III and IV were associated with increased PD-L1 IC expression as well as CD8^+^ effector genes but, compared with luminal cluster II, was associated with high PD-L1 TC expression. However, a reduced ORR was observed in basal subtypes compared with luminal cluster II subtype, suggesting that other immunosuppressive factors exist in the basal subtypes that prevent effective T-cell activation with inhibition of the PD-L1/PD-1 pathway [17]. The TCGA subtypes classification has to be included in the biomarker landscape in further clinical trials in order to understand the underlying immune biology to develop future rational combination or sequential treatment strategies. In the IMvigor211 trial, planned exploratory biomarker analyses included immune transcriptional gene expression (tGE) and tumor mutational burden (TMB). PD-L1 expression positively correlated with tGE (R = 0.61) but not TMB (R = 0.13). High PD-L1 and high tGE were associated with improved outcomes with both chemotherapy and atezolizumab. In contrast, higher TMB predicted OS only in favor of atezolizumab, appearing as a promising further biomarker for this agent [32]. Immune expression profiling improves the potential to accurately determine the inflammatory status of a tumor by quantifying chemokines, cytokines and cell surface proteins. In the Checkmate 275 study with nivolumab, a 25-gene IFN-γ signature was used to assess 177 tumor samples from pretreatment biopsies. Higher values in the IFN-γ gene signature were significantly correlated with response to nivolumab relative to low-value IFN-γ expression score (CR or PR in 20/59 patients with high IFN-γ signature relative to CR or PR in 19/118 patients with medium or low IFN-γ signature; p = 0.0003). Similar gene expression analysis performed with a chemokine panel showed enrichment in responses from patients with high expression of CXCL9 and CXCL10 demonstrating the potential to use gene expression profiling as a biomarker [19–33].

## Conclusion

Immunotherapy represents a milestone for patients with advanced urothelial carcinoma; different immune checkpoint inhibitors are currently available both in the second-line metastatic setting (after failure of platinum-based therapy) and in the first-line setting in cisplatin-ineligible patients. PD-L1 expression is not an ideal biomarker and further research is evaluating innovative methods to facilitate selection of patients who are most likely to benefit from these agents. Clinical trials are currently ongoing in order to evaluate new strategy such as combination of immunotherapy and chemotherapy or targeted agents.

## Future perspective

ICIs have now become a standard in mUC. However, only a proportion of patients respond to immunotherapy and progression occurs frequently in initially responding patients.

Combination of ICIs could appear promising, particularly the combination of PD-L1/PD-1 inhibitors and CTLA-4 inhibitors as their actions are complementary. While CTLA-4 is expressed by regulatory memory CD-4 and T cells and is functional during early activation of T cells in lymphatic tissues, PD-1 acts primarily during the effector phase of T-cell activation and the PD-1/PD-L1 interaction occurs primarily in peripheral tissues upon representation of antigens to memory T-cells.

As part of the CheckMate 032 trial, the combination of nivolumab plus ipilimumab was evaluated in pretreated patients with locally advanced or mUC, who had progressed on ≥1 prior lines of chemotherapy. Patients were treated with either of two combination schedules, nivolumab 1 mg/kg + ipilimumab 3 mg/kg (N1/I3) or nivolumab 3 mg/kg + ipilimumab 1 mg/kg (N3/I1) every 3 weeks for four cycles, followed by nivolumab 3 mg/kg every 2 weeks; or they were treated with nivolumab monotherapy 3 mg/kg (N3) every 2 weeks. A higher response rate was observed with N1/I3 (38.5%) compared with other cohorts (26% for N3/I1 and 25.6% for N3). Median DOR has not been reached in any treatment group. The median PFS in the N1/I3 group was 4.3 and 2.6 months in the N3/I1, with a median OS of 10.2 and 7.3 months, respectively. All-grade TRAEs were experienced by 76.9% of those in the N1/I3 arm compared with 84.6% of those in the N3/I1 arm. The rates of Grade 3/4 AEs were similar in each group, at 30.8 and 31.7%, for the N1/I3 and N3/N1 arms, respectively [34], compared with 28% observed in the nivolumab arm [20,21].

Other combinations are currently being evaluated. A Phase II trial (NCT01524991) is studying the association of gemcitabine, cisplatin, plus ipilimumab in chemotherapy-naive patients with mUC. 36 patients underwent two cycles of cisplatin–gemcitabine alone, followed by four cycles of gemcitabine, cisplatin and ipilimumab. The ORR was 64% with a median OS of 14.6 months, which seemed not superior to historical results of cisplatin–gemcitabine alone. This study thus did not meet the primary end point. In a translational analysis that included plasma collection for immunophenotyping, the addition of ipilimumab increased the proportion of CD4 and CD8 T-cells without depleting T-regulatory or myeloid-derived suppressor cells, suggesting the feasibility of this combination [35]. Ongoing studies will evaluate the benefit to associate ICIs with chemotherapy in mUC or in earlier stages.

The Phase II NCT02553642 is currently ongoing to evaluate the relationship between TMB and predicted neoantigen burden and response to nivolumab/ipilimumab in advanced BC.

**Executive summary**

- Immunotherapy is becoming a standard of care in metastatic urothelial carcinoma, improving survival outcome of patients.
- Five immune checkpoint inhibitors (ICIs; atezolizumab, nivolumab, pembrolizumab, avelumab and darvelumab) are approved by the US FDA as second-line agent after failure of platinum-based therapy. Pembrolizumab has a level 1 evidence in this setting as it was confirmed in a randomized Phase III clinical trial.
- In the first-line setting, for treatment of cisplatin-ineligible patients, pembrolizumab and atezolizumab represent reasonable choice and alternative to carboplatin regimen.
- Data concerning the correlation between ICIs efficacy and PD-L1 expression are heterogenous, and PD-L1 expression does currently not influence ICIs treatment.
- New biomarkers are currently evaluated including transcriptional gene expression, tumor mutational burden and immune expression profiling.
